# Primary prevention in chiropractic practice: a systematic review

**DOI:** 10.1186/s12998-017-0140-4

**Published:** 2017-03-20

**Authors:** Guillaume Goncalves, Christine Le Scanff, Charlotte Leboeuf-Yde

**Affiliations:** 1 CIAMS, University of Paris-Sud, University of Paris-Saclay, F- 91405 Orsay Cedex, France; 2 CIAMS, University of Orléans, F- 45067 Orléans, France; 3 Institut Franco Européen de Chiropraxie, 24 boulevard Paul Vaillant Couturier, F- 94200 Ivry sur Seine, France

**Keywords:** Chiropractic, Primary prevention, Public health, Prevention of musculoskeletal disorders, Wellness

## Abstract

**Background:**

Chiropractors are primarily concerned with musculoskeletal disorders but have the responsibility to deal also with prevention in other areas.

**Objectives:**

To establish the prevalence of chiropractors who have a positive opinion on the use of primary prevention (PP), their actual use of PP, and the proportion of patients who consult for PP in relation to (i) musculoskeletal disorders, (ii) public health issues, or (iii) chiropractic treatment for wellness.

**Method:**

A systematic search for literature was done using PubMed, Embase, Index to Chiropractic Literature, and Google Scholar and updated on February 15th 2017. Inclusion criteria were: surveys on chiropractors and/or chiropractic patients, information had to be present on PP in relation to the percentage of patients who consult for PP in chiropractic practice or in a chiropractic student clinic, and/or the percentage of chiropractors who reported using PP, and/or information on chiropractors’ opinions of the use of PP, in the English, French, or Scandinavian languages. The review followed the PRISMA guidelines. Articles were classified as ‘good’, ‘acceptable’ and ‘unacceptable’ based on scores of quality items. Results from the latter group were not taken into account.

**Results:**

Twenty-five articles were included, reporting on twenty-six studies, 19 of which dealt with wellness. The proportion of chiropractors who stated that they had a positive opinion on PP was generally higher than the proportion of chiropractors offering PP. Most chiropractors offered some type of PP for musculoskeletal disorders and more than a half stated that they did so in the public health area but also for wellness. For all types of PP, however, it was rarely stated to be the reason for patients consulting. Regardless the type of PP, the proportion of patients who actually consulted specifically for PP was much smaller than the proportion of chiropractors offering PP.

**Conclusion:**

More research efforts have been put into wellness than into prevention of musculoskeletal disorders or public health-related disorders. It therefore seems that parts of the chiropractic profession are in search of an understanding of various aspects of clinical practice over and above its traditional musculoskeletal role. Interestingly, only a small proportion of chiropractic patients consult for PP, despite the readiness of the profession to offer such services.

## Introduction

It is well accepted that non-communicable diseases, whether musculoskeletal or not, represent a social and economic burden, because they can be the source of long-term morbidity, and with increasing longevity they are expected to become increasingly common [1]. The prevention of such diseases can therefore minimize costs of health care, improve quality of life, and decrease both morbidity and mortality. Guidelines exist on how to approach this, such as the “Healthy People 2020”, which promotes modification of individual behaviour with a multidisciplinary approach [2].

Prevention can be performed at three stages of disease. Primary prevention (PP) deals with the prevention of disease in healthy people, secondary prevention is used to prevent a condition from recurring, whereas tertiary prevention is often defined as maintaining at a reasonable level a chronic condition that cannot be reversed [3]. In this review, we shall deal with PP only.

Chiropractors are recognized to be primary health care practitioners in many parts of the world, and consequently the regional Councils on Chiropractic Education state that a public health approach including health promotion should be implemented in chiropractic undergraduate programs [4–7]. It therefore seems logical that chiropractors have a role to play in the prevention of, at least, musculoskeletal disorders. Examples of this are campaigns in relation to posture, ‘Straighten up’ [8], and physical activity, ‘Just start walking’ [9].

Back pain and extremity problems can result in reduced physical activity with secondary consequences, such as obesity and reduced cardiovascular fitness, so the role of chiropractors would extend beyond that of trying to prevent back pain. In fact, the World Health Organization supports the concept that chiropractors have a role in the prevention of musculoskeletal disorders and other public health issues by stating that “Chiropractic is a health care profession concerned with the diagnosis, treatment and prevention of disorders of the musculoskeletal system and the effect of these disorders on general health” [10].

In addition to this, the World Federation of Chiropractic endorses and encourages chiropractors’ participation in public health promotion activities apart from musculoskeletal health [10]. Various preventive health-related issues, apart from the purely musculoskeletal, are also suitable to address in a primary care practice, some of which relate to life-style (e.g. nutrition, physical activities, and stress-management). The fact that chiropractic patients usually are partially undressed during examination and treatment makes also screening for skin cancers an appropriate task for chiropractors.

The ‘classical’ form of PP in relation to hygiene, improved working conditions, vaccinations etc. has resulted in large improvements of the public health status, but in more affluent countries and groups of people a more recent variant of PP has become apparent, that of the ‘wellness movement’. Wellness can be defined as “an active process in which an individual changes his or her behaviour in a manner which promotes health in all dimensions” [11]. Chiropractors, who traditionally adhere to the concept of healthy living, appear to have a natural inclination towards this approach.

Some chiropractors assume that a spinal derangement/dysfunction (variously called ‘subluxation’, ‘fixation’, ‘manipulative lesion’) can be reliably detected in both symptomatic and asymptomatic spines, and that the chiropractic manipulation (‘adjustment’), with or without other supportive treatments, can remove derangements and improve dysfunctions, a therapeutic approach which in turn is believed to have a favourable effect not only on present but also on future back problems. Some chiropractors also believe that this has a favourable effect on health in general, both in relation to a general feeling of well-being [12] and disease prevention [13]. Some even believe that this may impact on longevity [14, 15].

Some of the above preventive activities intuitively make sense, whereas others are controversial. Therefore, we wanted to learn more about what chiropractors think and do in relation to PP and also what actually happens in their clinic. In other words, do patients consult for PP? For these reasons, we undertook a systematic review to obtain answers to the following questions:

- What is the **prevalence of chiropractors** with positive opinions of the use of PP?
- What is the **prevalence of chiropractors** who use PP?
- What is the **proportion of chiropractic patients** who consult for PP?

We attempted to deal with each of these questions from three angles: 1/Musculoskeletal conditions, 2/Public health issues, and 3/Wellness, which we defined as PP through chiropractic care.

## Method

The AMSTAR checklist for methodological quality of systematic review [16] was followed except for assessment of publication bias and the assessment of conflict of interest, because there were no benefits to gain for surveying chiropractors. Also, we did not explicitly search the grey literature. The review was registered in PROSPERO (CRD42016049453).

### Search strategy

The search included peer-reviewed articles in journals that could be traced through PubMed, Embase, Index to Chiropractic Literature, and Google Scholar. We searched the literature from January 2000 until February 15th 2017 to include only recent information. Search strategies were developed with a health science research librarian, using free text words.

For Medline these were: “chiropract* **and** (wellness **or** primary **or** prevent* **or** health **or** promotion **or** service*) **and** (questionnaire* **or** survey*)”. In Embase the search strategy was: “chiropract* **and** (wellness **or** primary **or** prevent* **or** health **or** promotion **or** service*) **and** (questionnaire* **or** survey*) **and** [embase]/lim **not** [medline]/lim)”. In Index to Chiropractic Literature it was: “chiropract* **and** (wellness **or** primary **or** prevent* **or** health **or** promotion **or** service*) **and** (questionnaire* **or** survey*)”. In Google Scholar it was: “(chiropractic **or** chiropractors **or** chiropractor) **and** (wellness **or** primary **or** prevention **or** preventive **or** health **or** promotion **or** service **or** services) **and** (questionnaire **or** questionnaires **or** survey **or** surveys)”.

A hand search was also done consulting texts and reference lists of relevant articles. We did not search the non-peer reviewed literature specifically, but would accept such texts if they were easily available.

### Screening procedure

The first author (GG) selected the articles from the titles based on the inclusion and exclusion criteria. Thereafter, two authors (GG and CLY) independently screened abstracts and full texts using the inclusion and exclusion criteria.

Inclusion criteria were:

- Surveys on chiropractors and/or chiropractic patients.
- Information had to be present on: PP in relation to information on chiropractors’ opinions of the use of PP, and/or the percentage of chiropractors who reported using PP, and/or the percentage of patients who consult for PP in chiropractic practice or in a chiropractic student clinic.
- Languages: English, French, Swedish, Danish or Norwegian, as these were the languages the authors could easily read.

Exclusion criteria were:

- Articles reporting on the topics described above but on treatments not usually given by chiropractors (e.g. advice on vaccination, prevention in relation to stress/mental illness, orthopaedic shoes, substance abuse, injuries/trauma/falls/violence or non-muscular conditions in pregnant women). We also excluded articles on improvement of sport performance.
- If several publications existed from the same study, we would select the most relevant or complete of the publications in relation to our study objectives.

Chiropractic students and chiropractic academic staff were not defined as ‘chiropractors’.

### Data extraction

The information in the selected articles was reviewed in relation to two elements: 1/quality (i.e. representativeness and validity) and 2/results. Three checklists were designed for those aspects. Our requirements were lenient. We did not check contents of references to trace additional or missing information. We sought our information in the methods and result sections but not from the abstract or title.

A score was given to each selected article regarding various quality aspects and reported as a percentage. This score was used to determine the weak and strong points in this research field but also to classify the articles in descending order based on their individual total quality score. One point was given for correct answers. When the answer was incorrect or missing, it was given a score of 0. In some cases, half a score could be given. When an item was irrelevant because of the study design (e.g. no information would be available on patients if the purpose of the study was to study only chiropractors), it would be denoted as ‘irrelevant’.

The first checklist refers to the representativeness of study samples (Table 1). Points were given for the following reasons:

**Table 1:** The representativeness of twenty-six studies on the use of primary prevention in chiropractic practice

Articles 1st author Yr of publication Country of study	Study design in relation to our objectives i) data collected by DC ii) data collected by patients/ guardians	Target population defined (1 pt) i) DC ii) patients/guardians *Group(s) who provided the data were written in bold*	Study sample (s) described (at least age, sex, geographic distribution, or professional background) (1 pt) i) DC ii) patients/guardians	Sampling method -whole target population (1 pt) -random selection (1 pt) -consecutive sample (1 pt) -convenience sample (0 pt) i) DC ii) patients/guardians	Response rate provided or possible to calculate and if provided > 10% (1 pt) i) DC ii) patients/guardians	If less than 80% response, was there a resp/non-resp comparison? (1 pt) i) DC ii) patients/guardians	Scores
Walker (2000) [33] USA	i) DC report on their use of PP ii)/	i) American **DC** (1 pt) ii) IR	i) Yes (1 pt) ii) IR	i) Random selection (1 pt) ii) IR	i) 24% (1 pt) ii) IR	i) No (0 pt) ii) IR	4/5
Hawk (2001) [22] Australia Canada USA	i) DC report their use of PP and recruited patients to participate in survey ii) Patients report on RfC	i) **DC** in practice-based research network (1 pt) ii) DC’s **patients** (1 pt)	i) Yes (1 pt) ii) Yes (1 pt)	i) Convenience sample (0 pt) ii) Consecutive sampling (1 pt)	i) No (0 pt) ii) In a subsample response rate was estimated to be between 40 and 95% (1 pt)	i) No (0 pt) ii) No (0 pt)	6/10
Hawk (2004) [17] USA	i) DC report on their use of PP and opinions on PP ii)/	i) American **DC** (1 pt) ii) IR	i) Yes (1 pt) ii) IR	i) Random selection (1 pt) ii) IR	i) 27% (1 pt) ii) IR	i) No (0 pt) ii) IR	4/5
McDonald (2004) [34] Mexico USA Canada	i) DC report on their opinions on PP ii)/	i) **DC** from mainly North America (1 pt) ii) IR	i) Yes (1 pt) ii) IR	i) Random selection (1 pt) ii) IR	i) 63% (1 pt) ii) IR	i) Yes (1 pt) ii) IR	5/5
Mootz (2005) [38] USA	i) DC collected data on their patients' RfC ii)/	i) American **DC** from Arizona and Massachusetts (1 pt) ii) DC's patients (1 pt)	i) Yes (1 pt) ii) Yes (1 pt)	i) Random selection (1 pt) ii) Consecutive sampling (1 pt)	i) 68% (Arizona) 76% (Massachusetts) (1 pt) ii) 58% (Arizona) 67% (Massachusetts) (1 pt)	i) Yes (1 pt) ii) Yes (1 pt)	10/10
Alcantara (2008) [23] Several countries	i) DC collected data on their patients' RfC and recruited patients to participate in survey ii) Patients report on RfC	i) **DC** in practice-based pediatric research network (1 pt) ii) Parents of DC's **patients** (1 pt)	i) No (0 pt) ii) Yes (1 pt)	i) Convenience sample (0 pt) ii) Not reported (0 pt)	i) 2% (0 pt) ii) No (0 pt)	i) No (0 pt) ii) No (0 pt)	3/10
Blum (2008) [18] Australia Europe USA	i) DC recruited patients to participate in survey ii) Patients report on RfC	i) DC specialized in SOT and known to use wellness (1 pt) ii) DC's **patients** (1 pt)	i) No (0 pt) ii) Yes (1 pt)	i) Convenience sample (0 pt) ii) Consecutive sample (1 pt)	i) 100% (1 pt) ii) No (0 pt)	i) NA because >80% (1 pt) ii) No (0 pt)	6/10
Malmqvist (2008) [35] Finland	i) DC report on their use of PP ii)/	i) **DC** from Finland (1 pt) ii) IR	i) Yes (1 pt) ii) IR	i) Whole population (1 pt) ii) IR	i) 88% (1 pt) ii) IR	i) NA because >80% (1 pt) ii) IR	5/5
Alcantara (2009) [24] Several countries	i) DC report on patients’ RfC ii) Patients report on RfC	i) **DC** in practice-based pediatric research invited the patients and were also surveyed (1 pt) ii) Parents of DC's **patients** (1 pt)	i) No (0 pt) ii) Yes (1 pt)	i) Convenience sample (0 pt) ii) Not reported (0 pt)	i) 1% (0 pt) ii) No (0 pt)	i) No (0 pt) ii) No (0 pt)	3/10
Hestbaek (2009) [37] Denmark	i) DC recruited patients to participate in survey ii) Patients report on RfC	i) Danish DC treating pediatric patients (1 pt) ii) Pediatric **patients** after their 1st visit (1 pt)	i) No (0 pt) ii) Yes (1 pt)	i) Whole population (1 pt) ii) Consecutive sample of new patients (1 pt)	i) 84% (1 pt) ii) No probably > 50% (0 pt)	i) NA because >80% (1 pt) ii) Yes? (1 pt)	8/10
Alcantara (2010) [25] Several countries	i) DC report on their use of PP and patients’ RfC ii)/	i) **DC** in practice-based pediatric research network (1 pt) ii) Pediatric patients (1 pt)	i) Yes (1 pt) ii) No (0 pt)	i) Convenience sample (0 pt) ii) Not reported (0 pt)	i) 37% (1 pt) ii) No (0 pt)	i) No (0 pt) ii) No (0 pt)	4/10
Leach (2011) [28] USA	i) DC report on their opinions on PP and use of PP ii)/	i) **DC** in state of Mississippi (1 pt) ii) IR	i) Yes (1 pt) ii) IR	i) Whole population (1 pt) ii) IR	i) 43% (1 pt) ii) IR	i) No (0 pt) ii) IR	4/5
Marchand (2012) [26] Several European countries	i) DC report on their use of PP and collect data on their patients' RfC ii)/	i) **DC** from several European countries (1 pt) ii) DC's patients (1 pt)	i) Yes (1 pt) ii) No (0 pt)	i) Whole population (1 pt) ii) Not reported (0 pt)	i) 23% (1 pt) ii) IR	i) No (0 pt) ii) IR	5/8
French (2013) [39] Australia	i) DC collect data on their patients' RfC ii)/	i) Australian **DC** (1 pt) ii) Patients from these DC (1 pt)	i) Yes (1 pt) ii) Yes (1 pt)	i) Random selection (1 pt) ii) Consecutive sample (1 pt)	i) 33% (1 pt) ii) 86% (1 pt)	i) No (0 pt) ii) NA because >80% (1 pt)	9/10
Stuber (2013) [19] Canada	i) DC report on their use of PP ii)/	i) **DC** from the province of Saskatchewan (1 pt) ii) IR	i) Yes (1 pt) ii) IR	i) Whole population (1 pt) ii) IR	i) 45% (1 pt) ii) IR	i) Yes (1 pt) ii) IR	5/5
Brown (2014) [40] Australia	i) DC recruit patients to participate in survey ii) Patients report on their opinions on PP	i) Australian chiropractic clinics (1 pt) ii) Adult **patients** from these clinics (1 pt)	i) No (0 pt) ii) Yes (1 pt)	i) Random selection (1 pt) ii) Consecutive sample (1 pt)	i) 96% (1 pt) ii) 24% (1 pt)	i) NA because >80% (1 pt) ii) No (1 pt)	9/10
McGregor (2014) [20] Canada	i) DC report on their opinions on PP ii)/	i) English speaking Canadian **DC** (1 pt) ii) IR	i) Yes (1 pt) ii) IR	i) Random selection (1 pt) ii) IR	i) 68% (1 pt) ii) IR	i) No (0 pt) ii) IR	4/5
Bussières (2015) [27] Canada	i) DC report on their opinions on PP ii)/	i) Canadian **DC** with a valid email address (1 pt) ii) IR	i) Yes (1 pt) ii) IR	i) Convenience sample (0 pt) ii) IR	i) 8% (0 pt) ii) IR	i) No (0 pt) ii) IR	2/5
Blanchette (2015) [36] Canada	i) DC report on their opinions on PP ii)/	i) Canadian **DC** (1 pt) ii) IR	i) Yes (1 pt) ii) IR	i) Whole population (1 pt) ii) IR	i) 39% (1 pt) ii) IR	i) Yes (1 pt) ii) IR	5/5
Fikar (2015) [31] UK	i) DC report on their opinions on PP and use of PP ii)/	i) English **DC** (1 pt) ii) IR	i) Yes (1 pt) ii) IR	i) 4 Whole populations (1 pt) ii) IR	i) 21% (1 pt) ii) IR	i) No (0 pt) ii) IR	4/5
Glithro (2015) [29] UK	i) DC report on their opinions on PP and use of PP ii)/	i) English **DC** including some students (1 pt) ii) IR	i) Yes (1 pt) ii) IR	i) Random selection (1 pt) ii) IR	i) 30% (1 pt) ii) IR	i) No (0 pt) ii) IR	4/5
Schneider (2015) [30] USA	i) DC report on their opinions on PP ii)/	i) American **DC** (1 pt) ii) IR	i) Yes (1 pt) ii) IR	i) Convenience sample (0 pt) ii) IR	i) maximum 4% (0 pt) ii) IR	i) No (0 pt) ii) IR	2/5
Allen- Unhammer (2016) [21] Norway (Part1 – register study)	i) DC report on their patients’ RfC in NHS database ii)/	i) Norwegian **DC** (1 pt) ii) Paediatric patients from these DC (1 pt)	i) No (0 pt) ii) Yes (1 pt)	i) Whole target population (1 pt) ii) Whole target population (1 pt)	i) NA (register data) Probably 100% (1 pt) ii) NA (register data) Probably 100% (1 pt)	i) NA because >80% (1 pt) i) NA because >80% (1 pt)	9/10
Allen- Unhammer (2016) [21] Norway (Part 2 – survey)	i) DC recruit paediatric patients ii) patients/parents report on RfC	i) Norwegian **DC** (1 pt) ii) Paediatric patients from these DC (1 pt)	i) Yes (1 pt) ii) Yes (1 pt)	i) Whole target population (1 pt) ii) Convenience sample from small group of participating DC (0 pt)	i) 15% (1 pt) ii) No (0 pt)	i) No (0 pt) ii) No (0 pt)	6/10
Pohlman (2016) [41] Several Countries	i) DC report on their patients’ RfC ii)/	i) **DC** (1 pt) ii) IR	i) Yes (1 pt) ii) IR	i) 3 whole populations (1 pt) ii) IR	i) 29% (1 pt) ii) IR	i) Yes (1 pt) ii) IR	5/5
Adams (2017) [32] Australia	i) DC report on their use of PP ii)/	i) Australian **DC** (1 pt) ii) IR	i) yes (1 pt) ii) IR	i) Whole target population (1 pt) ii) IR	i) 43% (1 pt) ii) IR	i) No (0 pt) ii) IR	4/5

*PP* Primary Prevention, *DC* chiropractors, *IR* irrelevant, *NA* Not Applicable

*RfC* Reason for Consulting, *NHS* National Health Service

- **Target population defined**: Specific subpopulations may have different practice patterns, therefore it is important to define the target population. This would give one point.
- **Study sample**: One point was given if the study sample(s) was/were described at least for age, sex, geographical distribution, or professional background.
- **Sampling method**: To avoid selection bias, the whole population, a random, or – possibly – a consecutive sample would be needed, resulting in one point, whereas a convenience sample brought 0 points. National chiropractic associations were considered whole populations and conference participants were classified as belonging to a convenience sample.
- **Response rate**: The higher the response rate, the easier to generalize the results to the underlying population. Therefore, the reader needs to be informed of the percentage of participants. One point was given for providing this information or if it was possible to calculate. Response rates in surveys are often low but, nevertheless, we considered samples of 10% or less to be unacceptable, resulting in 0 point, as it would severely limit the generalisability of the results in such cases.
- **Response/Non response comparison**: If the response rate was lower than the arbitrarily determined cut-point of 80%, we expected to find some type of responder/non-responder analysis. One point was given for this, if this comparison was needed. If it was not needed, because the response rate was above this cut-point, the response was defined as “not applicable” and given one point as well. If the response rate was not given but a response/non response comparison done, one point was given for the latter but not for the former.

The second checklist deals with the validity of the results (Table 2). Points were considered for the following items:

**Table 2:** The validity of twenty-six studies on the use of primary prevention in chiropractic practice

Articles 1st author Yr of publication Country of study	If prevention studied as main topic, was there a definition/explanation (in the introduction or the method)? (1 pt)	Were relevant questions or survey instrumenprovided? (1 pt)	Was there an attempt to assure quality of survey instrument?	DC attitudes and use		Reasons for consulting		Scores
				Opinions to PP -anonymous reporting/confidentiality (1 pt)	Use of PP -anonymous reporting/confidentiality (1 pt)	Reported by DC -actuarial reporting (1 pt) -approx. reporting (0 pt)	Reported by patient/ guardians -anonymous reporting/confidentiality (1 pt)	
Walker (2000) [33] USA	IR	No (0 pt)	Yes (1 pt)	IR	Not reported (0 pt)	IR	IR	1/3
Hawk (2001) [22] Australia Canada USA	IR	No (0 pt)	Yes (1 pt)	IR	Not reported (0 pt)	IR	Yes (1 pt)	2/4
Hawk (2004) [17] USA	Yes “public health, clinical prevention, or health promotion” (1 pt)	No (0 pt)	Yes (1 pt)	Yes (1 pt)	Yes (1 pt)	IR	IR	4/5
McDonald (2004) [34] Mexico USA Canada	IR	No (0 pt)	Yes (1 pt)	IR	Not reported (0 pt)	IR	IR	1/3
Mootz (2005) [38] USA	IR	Available on request (1 pt)	Yes (1 pt)	IR	IR	Actuarial reporting (1 pt)	IR	3/3
Alcantara (2008) [23] Several countries	IR	No (0 pt)	Yes (1 pt)	IR	IR	Actuarial reporting (1 pt)	Yes (1 pt)	3/4
Blum (2008) [18] Australia Europe USA	Yes *Wellness*: Optimizing health among self-identified healthy. *Prev. Health*: 1/Preventing illness among self-identified healthy 2/Preventing illness in people at risk (1 pt)	Yes (1 pt)	No (0 pt)	IR	IR	IR	Yes (1 pt)	3/4
Malmqvist (2008) [35] Finland	IR	Yes (1 pt)	Yes (1 pt)	IR	Yes (1 pt)	IR	IR	3/3
Alcantara (2009) [24] Several countries	IR	No (0 pt)	Yes (1 pt)	IR	IR	Actuarial reporting (1 pt)	Yes (1 pt)	3/4
Hestbaek (2009) [37] Denmark	IR	No (0 pt)	Yes (1 pt)	IR	IR	IR	Yes	2/3
Alcantara (2010) [25] Several countries	IR	No (0 pt)	Yes (1 pt)	IR	Yes (1 pt)	Approximate reporting (0 pt)	IR	2/4
Leach (2011) [28] USA	Yes Refers to ‘’Healthy people 2010” (1 pt)	Yes (1 pt)	Yes (1 pt)	Yes (1 pt)	Yes (1 pt)	IR	IR	5/5
Marchand (2012) [26] Several European countries	IR	No (0 pt)	Yes (1 pt)	IR	IR	Approximate reporting (0 pt)	IR	1/3
French (2013) [39] Australia	IR	Yes (1 pt)	Yes (1 pt)	IR	IR	Actuarial reporting (1 pt)	IR	3/3
Stuber (2013) [19] Canada	IR	No (0 pt)	Yes (1 pt)	IR	Yes (1 pt)	IR	IR	2/3
Brown (2014) [40] Australia	IR	No (0 pt)	Yes (1 pt)	IR	IR	IR	Yes (1 pt)	2/3
McGregor (2014) [20] Canada	IR	Yes (1 pt)	Yes (1 pt)	Yes (1 pt)	IR	IR	IR	3/3
Bussières (2015) [27] Canada	IR	No but very informative tables (0,5*pt)	Yes (1 pt)	Yes (1 pt)	IR	IR	IR	2.5/3
Blanchette (2015) [36] Canada	IR	No, but very informative tables (0,5*pt)	No (0 pt)	IR	Not reported (0 pt)	IR	IR	0.5/3
Fikar (2015) [31] UK	Yes ‘’Promote health and wellness” (1 pt)	No (0 pt)	No (0 pt)	Probably yes (1 pt)	Probably yes (1 pt)	IR	IR	3/5
Glithro (2015) [29] UK	Yes ‘’Early detection of pre-cancerous lesion” (1 pt)	No, but very informative tables (0,5*pt)	Yes (1 pt)	Yes (1 pt)	Yes (1 pt)	IR	IR	4.5/5
Schneider (2015) [30] USA	IR	Yes (1 pt)	Yes (1 pt)	Yes (1 pt)	IR	IR	IR	3/3
Allen- Unhammer (2016) [21] Norway (Part1 – register study)	IR	IR	IR	IR	IR	Actuarial reporting (1 pt)	IR	1/1
Allen- Unhammer (2016)[21] Norway (Part 2 – survey)	IR	No (0 pt)	Yes (1 pt)	IR	IR	IR	Actuarial reporting (1 pt)	2/3
Pohlman (2016) [41] Several Countries	IR	No (0 pt)	Yes (1 pt)	IR	IR	Approximate reporting (0 pt)	IR	1/3
Adams (2017) [32] Australia	IR	No (0 pt)	Yes (1 pt)	Not reported (0 pt)	IR	IR	IR	1/3

PP: Primary Prevention/DC: chiropractors/IR: irrelevant

- **Definition/explanation of PP**: PP must be well defined or at least explained in order to show that the authors have a clear understanding of which concept they are studying. However, it was not considered reasonable to expect authors to define every aspect of a study with multiple outcome variables. Therefore, this definition was required only if prevention was the main topic of the study (one point if there was a definition in the introduction or method in articles having prevention as main topic).
- **Relevant questions or questionnaires available for the reader**: Questions and/or questionnaires must be appropriate, for which reason it is important to make them accessible in the article or available on request, thus resulting in one point.
- **Attempt to assure quality of survey instrument**: The quality of the survey instrument was considered acceptable if questions were selected based on a thorough review of the literature, if there was a pilot study, or if the questionnaire/relevant questions had been previously tested at least for user friendliness, thus resulting in one point.
- **Opinions to PP, as reported by chiropractors**: One point was given if the reporting was anonymous, or if the confidentiality of the chiropractor was respected.
- **Use of PP**: One point was given if the reporting was anonymous or if the confidentiality of the chiropractor was respected.
- **Reasons for consulting reported by the chiropractor**: One point was given for actuarial reporting (i.e. file search or actual counting) and 0 point for approximate reporting (i.e. based on non-factual information).
- **Reasons for consulting reported by patients**: One point was given for patients providing reasons for consulting independently of the treating chiropractors (anonymously) or if it was stated that the patients’ confidentiality was respected.

One of the authors of this review had co-authored one of the reviewed articles, therefore a third person reviewed that article. Disagreements between the two reviewers were discussed to achieve consensus. If they could not reach agreement, the third author would be consulted.

Thereafter, articles were arbitrarily classified, based on the scores of the two quality checklists. The article was classified as ‘good’ if the final score was ≥ 80%, as ‘acceptable’ if the final score was between 60 and 79%, and as ‘unacceptable’ if the final score was < 60%. This classification was partly based on the spread of data, because the difference between groups, particularly between ‘acceptable’ and ‘unacceptable’, should not depend on one single point.

It was often difficult to understand how chiropractors and patients defined the three concepts of PP (prevention of musculoskeletal disorders, public health prevention, wellness through chiropractic treatment). In such cases, we looked for specific words in the text that could indicate the underlying meaning and classified the articles as shown in Table 3.

**Table 3:** Words used to determine type of primary prevention studied in chiropractic practice

Prevention of musculoskeletal disorders	Public health prevention	Wellness through chiropractic care including spinal adjustments^a^
Ergonomic advice	Physical activity	Wellness
Postural advice/improvements	General health	Prevention in children
Prophylactic exam	Health enhancement	General well being
Prevention, if not described under Public health or wellness	At risk	Subluxation
	Recommendations/advice on health issues	
	Nutritional and dietary advice	

^a^Unless explicitly stated that “wellness” and other words in column 3 relate to advice only, it was be assumed that it had an element of chiropractic adjustments (with or without advice)

### Analysis and presentations of data

Assessment of the articles was done using the checklists independently by two of the authors, after which their respective checklists were compared, followed by a discussion on unclear points. Such queries were always resolved, because usually different interpretations of articles arose from difficulties in finding the relevant text.

The articles were arranged in descending order in relation to their classification and their final quality score with a colour-coding of the three subgroups (i.e. ‘good’, ‘acceptable’, and ‘unacceptable’). Results (Table 4) were thereafter interpreted for each of the three main concepts of PP (musculoskeletal, public health and wellness) in relation to the three main study objectives of the study. When interpreting the results we disregarded the studies that we considered to be of unacceptable quality. For the others, if estimates of similar items were largely different, mainly studies with the better-quality scores would be taken into account. Therefore, results were first considered for the ‘good’ studies and then for the ‘acceptable’ studies.

**Table 4:** The results in twenty-three articles on the use of primary prevention in chiropractic practice

Articles 1st author Yr of publication Country of study [Quality rating]	Chiropractors’ positive opinions on PP			Chiropractors’ use of PP			Patients’ reason for consulting (RfC)		
	MSK prevention	General public health approach to PP unrelated to adjustments	Wellness likely to include adjustments	MSK prevention	General public health approach to PP unrelated to adjustments	Wellness likely to include adjustments	MSK prevention	General public health approach to PP unrelated to adjustments	Wellness likely to include adjustments
	1	2	3	4	5	6	7	8	9
Mootz (2005) [38] USA GOOD [100%]									Primary RfC: 4% ‘wellness’ (Arizona) 10% ‘wellness’ (Massachusetts)
Malmqvist (2008) [35] Finland GOOD [100%]						48% use ‘wellness’			
French (2013) [39] Australia GOOD [92%]									RfC: 6% for ‘health maintenance or preventive care’
Allen- Unhammer (2016) [21] Norway GOOD [91%]							RfC: 1% for ‘prophylactic examination’		
Leach (2011) [28] USA GOOD [90%]		94% positive to physical activity prescription 66% on tobacco cessation advice *See Table* *6*	92% were ‘wellness-oriented’		86% prescribed physical activity or advised on this topic 60% advised on tobacco cessation *See Table* *6*				
Stuber (2013) [19] Canada GOOD [87%]					82% ‘’recommend dietary supplements (…) for general health and wellness”				
McGregor (2014) [20] Canada GOOD [87%]			19% thought chiropractic subluxation is an obstruction to human health						
McDonald (2004) [34] Several countries GOOD [85%]						94% included periodic MC/wellness care in their clinical routine			
Brown (2014) [40] Australia GOOD [85%]									RfC: 21% for ‘general health and well-being’
Glithro (2015) [29] UK GOOD [85%]		81%* agreed that screening patients for skin lesions was part of their clinical role *Includes some DC students			*Skin lesions*: −94% screened each new patient −53% screened regular patients at every visit −73% screened regular patients at visits scheduled specifically for patient re-assessment.				
Hawk (2004) [17] USA GOOD [80%]		91% positive to nutritional advice 95% on the prescription of physical activity 69% on tobacco cessation advice 57% on skin lesion screening *See Table* *6*	93% had a positive attitude to subluxation screening	90% of chiropractors provide information on MSK risk reduction	86% gave nutritional advice 89% prescribed physical activity or advised on this topic 65% advised on tobacco cessation 46% screened for skin lesion *See Table* *6*				
Hestbaek (2009) [37] Denmark ACCEPTABLE [77%]							RfC: 7% for ‘prophylactic examination’		RfC: 2% for ‘general well being’
Pohlman (2016) [41] Several countries ACCEPTABLE [75%]									RfC: 18% for wellness
Fikar (2015) [31] UK ACCEPTABLE [70%]		62 to 97% considered lifestyle issues to be their responsibility to discuss		96% advised on poor posture 88% advised on ‘faulty movement patterns’	79% gave nutritional advice 92% prescribed physical activity or advised on this topic 57% advised on tobacco cessation *See Table* *6*				
Blanchette (2015) [36] Canada ACCEPTABLE [69%]						For 59% of patients Maintenance/ Wellness was the main sector of activity			
Blum (2008) [18] Several countries ACCEPTABLE [64%]							RfC in asymptomatic patients: 12% for ‘prevention’ *See Table* *5*	RfC in asymptomatic patients: 16% for being ‘at risk’ *See Table* *5*	RfC in asymptomatic patients: 14% for ‘wellness’ *See Table* *5*
Walker (2000) [33] USA ACCEPTABLE [62%]					77% used nutrition for ‘general healthful eating/nutrition’				
Schneider (2015) [30] USA ACCEPTABLE [62%]			8% focused on ‘wellness/ prevention’						
Allen- Unhammer (2016) [21] Norway (Part 2 – survey) ACCEPTABLE [62%]							RfC: <5% for infants <3 mo <10% for infants 4–23 mo ‘prophylactic examination’		
Adams (2017) [32] Australia ACCEPTABLE [62%]				73% treated patients for ‘spinal health maintenance/prevention’.					
Hawk (2001) [22] Several countries UNACCEPTABLE [57%]					48% used ‘diet/nutrition counselling for general health’ 46% used ‘exercise counseling’			RfC: <1% for disease prevention/health promotion through nutrition	RfC: 3% for disease prevention/health promotion through ‘subluxation correction’
Bussières (2015) [27] Canada UNACCEPTABLE [56%]			9% focused on ‘wellness/ prevention’						
Marchand (2012) [26] Several countries UNACCEPTABLE [55%]							RfC: <1% for ‘posture screening Prevention’		RfC: <1% for ‘advice/check up birth check up’ Wellness
Alcantara (2008) [23] Several countries UNACCEPTABLE [43%]									*RfC reported by DC* 35% were reported as ‘wellness care’ *RfC reported by patients* 44% of parents gave ‘wellness care’ as the motivation to consult
Alcantara (2009) [24] Several countries UNACCEPTABLE [43%]									*RfC reported by patients* 35% ‘were reported as presenting for wellness care’ *RfC reported by patients* 47% ‘presented for wellness care’
Alcantara (2010) [25] Several countries UNACCEPTABLE [43%]						90%: used ‘wellness care’	RfC: 2% of DC have patients who consult for ‘postural improvement’		RfC: 17% of DC have patients who consult for ‘wellness care’

MC: Maintenance Care/RfC: Reasons for Consulting/DC: chiropractors

## Results

### Description of studies

**Fig. 1:**
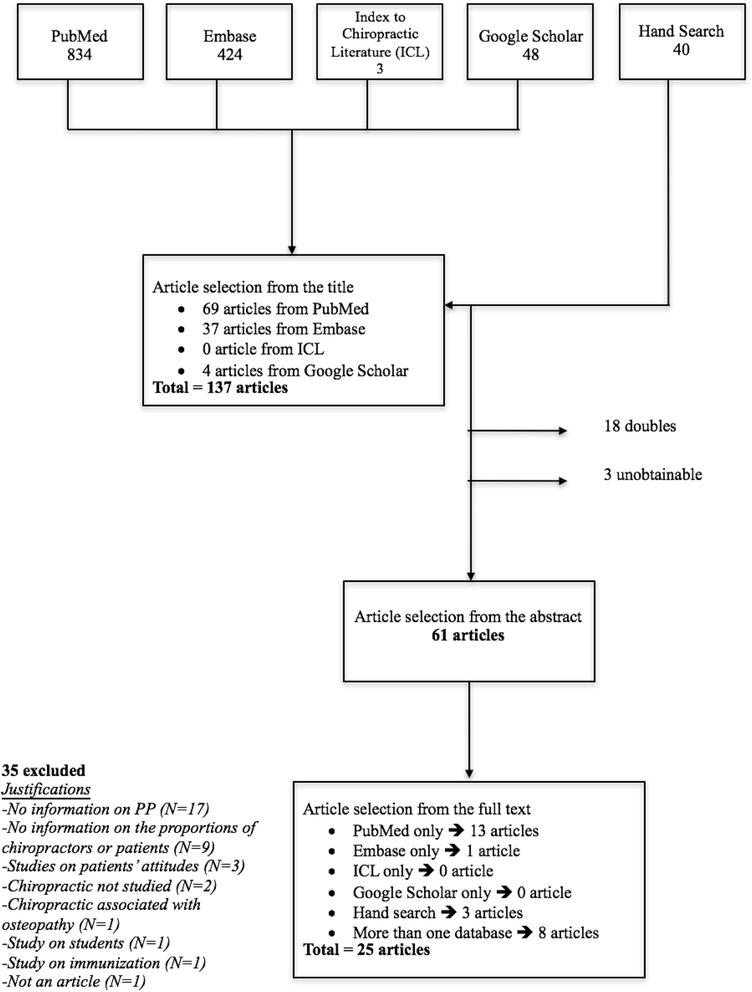
Description of the search for literature in a review of primary prevention in chiropractic practice

As can be seen in Fig. 1, of the 1349 initially screened articles, we retained 25 that were published between 2000 and 2017. Five of these studied prevention as their main topic and all of these attempted to describe what was meant by PP. One of these stood out by using a particularly complete definition of prevention in relation to the level of perceived health in the target group (Table 5). One of the studies dealt with the early detection of pre-cancerous lesions, whereas words such as public health, health promotion, wellness, preventing illness, and ‘Healthy People’ were used in the others. Nevertheless, clearly specific definitions were rarely provided. When ‘wellness’ was the topic (*n* = 19), a description of how exactly it was perceived or dealt with, was provided only in four articles [17–20]. One article [21] reported on two separate studies of different design that were reported as such in tables and text.

**Table 5:** An example of a definition of primary prevention from the point of view of patients

Behavior	Definition of primary prevention
Wellness	Activity undertaken by a person, who believes himself to be healthy, for the purpose of attaining a greater level of health.
Preventive Health	Activity, undertaken by a person, who perceives himself to be healthy, for the purpose of preventing illness or detecting it in an asymptomatic state.
At-risk	Activity undertaken by a person, who believes himself to be developing a specific health condition, for the purpose of preventing that condition or detecting it in an asymptomatic state.

Modified text taken from Handbook of Clinical Chiropractic Care. 2005: Jones and Barlett Publishers, Sudbury, MA. www.jbpub.com

As shown in Table 4, chiropractors’ use or opinions of PP were studied in 15 studies and their patients were targeted in 13 of the studies. Nine studies dealt with specific chiropractic interest groups, such as those specializing in paediatric treatment (*n* = 7).

When chiropractors were the source of information on PP, seven studies reported on their opinions about PP in their practice, and the prevalence of chiropractors using PP was reported also in 12 studies. Nineteen of the studies dealt with PP in relation to wellness, eight discussed PP in the light of public health, and nine concerned themselves with the PP of musculoskeletal conditions.

Eleven studies were classified as ‘good’, nine as ‘acceptable’, and six as ‘unacceptable’ in relation to their methodological quality. As shown in Tables 1 and 2, the least frequently covered methodological items were 1/an appropriate responder/non responder analysis (missing 22 times/37 possible), 2/the provision of relevant questions or survey instrument (missing 15 times/25), 3/an appropriate sampling method (missing 12 times/38). Six articles [22–27], considered by us to be ‘unacceptable’ (four reporting on paediatric subgroups), were ignored in the data analysis based on our pre hoc decision. The scores in each study have been incorporated in the result checklist (Table 4).

The many public health attitudes and activities reported in the various studies were listed but not described in Table 6. Only five of these topics were arbitrarily selected for our analysis (Table 4). These were: (i) prescription of dietary supplements or advice on nutrition; (ii) prescription of/advice on physical activity; (iii) advice on tobacco cessation; (iv) detection of skin lesion; and (v) non-specific public health). They seem best to represent the opinions and actions of the surveyed chiropractors in relation to their public health approach.

**Table 6:** All reported attitudes and activities in relation to public health in surveys on chiropractic practice

Examples of PP	Leach (2011) [28] USA		Stuber (2013) [19] Canada		Glithro (2015) [29] UK		Hawk (2004) [17] USA		Fikar (2015) [31] UK		Walker (2000) [33] USA		Adams (2016) [32] Australia		Hawk (2001) [22] Several	
	Opi	Use	Opi	Use	Opi	Use	Opi	Use	Opi	Use	Opi	Use	Opi	Use	Opi	Use
Prescription of dietary supplements or advice on nutrition				X			X	X		X		X				X
Prescription of physical activity or advice on this topic	X	X					X	X		X						X
Tobacco cessation advice	X	X					X	X		X				X		
Detection of skin lesion					X	X	X	X								
Advice on substance abuse	X	X					X	X								
Advice on responsible sexual behaviour	X	X					X	X								
Advice on alcohol abuse/dependence							X	X	X					X		
Advice on traffic security							X	X								
Advice on domestic violence							X	X								

*Opi* opinions, *Use*: use of service

### What is the prevalence of chiropractors with positive opinions on the use of PP?

#### Musculoskeletal disorders (Table 4, column 1)

There was no study reporting on chiropractors’ opinions on musculoskeletal PP.

#### General public health approach (Table 4, column 2)

Two ‘good’ studies [17, 28] reported on chiropractors’ opinions on PP for public health in general, showing that the vast majority of chiropractors (around 90%) had positive opinions on the prescription of physical activity or nutritional advice. Also, almost 70% of chiropractors had positive opinions on tobacco cessation advice. The proportion of chiropractors who had positive opinions on skin lesion detection varied between 57% and 81% [17, 29], depending on how the question was asked.

#### Wellness (Table 4, column 3)

Two studies (one ‘good’, one ‘acceptable’) reported positive opinions on ‘wellness’, without further definitions or explanations. In the ‘good’ article [28], 92% of chiropractors were reported to be “wellness-oriented” whereas in the other, 8% agreed to being focused on “wellness/prevention” [30].

Two other ‘good’ surveys defined wellness through the treatment of spinal ‘subluxation’. According to one of them, 19% of chiropractors considered the “chiropractic subluxation as an obstruction to human health” (by the author of that article these chiropractors were classified as ‘unorthodox’) [20], whereas, according to the second study, 93% of chiropractors had a positive attitude to ‘subluxation screening’, which could include several types of prevention but, in our opinion, indicated a belief in the use of subluxation detection as part of PP [17].

### What is the prevalence of chiropractors who use primary prevention?

#### Musculoskeletal disorders (Table 4, column 4)

Three studies dealt with PP of musculoskeletal disorders. According to the ‘good’ study, 90% of chiropractors provided information on prevention of musculoskeletal disorders [17].

One of two ‘acceptable’ studies was in agreement with the ‘good’ one, with similar high percentages for advice on posture (96%) and movement patterns (88%) [31]. The other ‘acceptable’ study [32] reported that more than 70% of chiropractors treated patients for ‘spinal health maintenance/prevention’, without specifying the type of prevention (primary or other).

#### General public health approach (Table 4, column 5)

Seven articles dealt with public health advice and public health screening procedures included in chiropractic consultations. All of these articles reported on the use of various screening procedures and lifestyle advice.

Lifestyle advice reported in relation to nutrition was dealt with in four studies (two ‘good’ and two ‘acceptable’). The two ‘good’ [17, 19] articles reported that 86% and 82% of chiropractors give nutritional advice in their practice. The other two studies [31, 33] reported this for 77% and 79%.

Chiropractors also reported that they prescribed or advised on physical activity. According to three articles (two ‘good’ [17, 28], one ‘acceptable’ [31]), around 90% of chiropractors did this type of PP. All of these three articles dealt also with tobacco cessation and reported that around 60% of chiropractors gave advice on that subject.

Two ‘good’ articles dealt with the screening for skin cancers. One reported that about 50% of chiropractors did this type of prevention, without defining the frequency of use [17]. The other article [29] reported the same proportion (53%) for the chiropractors who did this prevention at every visit, and showed that 94% screened all new patients.

One ‘acceptable’ article [32] dealt with ‘smoking/drug/alcohol’. It was impossible to isolate data on smoking cessation only, the prevalence of chiropractors using this global lifestyle approach was therefore not included in Table 4.

#### Wellness (Table 4, column 6)

One ‘good’ study [34] reported that more than 90% of chiropractors included periodic maintenance care/wellness care in their clinical routine. This means that the exact proportion of PP is unknown, as maintenance care would be a mixture of secondary and tertiary prevention.

Two studies reported the use of wellness without further specification. It was used by approximately 50% of chiropractors according to both the ‘good’ [35] and the ‘acceptable’ [36] study. The ‘acceptable’ study also included maintenance care under the definition of wellness, as chiropractors’ main sector of activity, thus – again – making it impossible to differentiate between the two.

### What is the proportion of chiropractic patients who consult for primary prevention?

#### Musculoskeletal disorders (Table 4, column 7)

Four studies (one ‘good’ [21], three ‘acceptable’ [18, 21, 37]) informed us about the proportion of patients who consulted for prevention of musculoskeletal disorders. One [18] of the ‘acceptable’ studies dealt with the general population. The other three, two of which were reported in one article, dealt with paediatric patients [21, 37]. The proportion of patients who consulted for PP was around 10% in all ‘acceptable’ studies. However, the ‘good’ study, which in fact based its data on all chiropractic consultations in Norway during a given period, reported a proportion of only 1%.

#### General public health approach (Table 4, column 8)

One’acceptable’ article dealt with the aspect of PP through a classical public health concept, by asking patients for their reasons to consult. In this study of chiropractic patients consulting practitioners with a special interest in wellness, 16% [18] considered themselves to be at risk. For an explanation of this concept, see Table 5.

#### Wellness (Table 4, column 9)

When patients came for a ‘wellness consultation’ it was difficult to know what they really aimed for. In three ‘good’ [38–40] and three ‘acceptable’ [18, 37, 41] studies, none made it perfectly clear that by ‘wellness’ they meant disease prevention through ‘subluxation correction’. Nevertheless, in these studies the chiropractors were said to be primarily consulted for ‘wellness’ and/or ‘preventive care’, and it seems unlikely that patients would primarily consult the chiropractor to provide preventive work other than through ‘classical’ chiropractic care (i.e. spinal manipulation and other usual, associated activities). The prevalence for this ranged between 2% (paediatric patients) to 21% (adult patients).

## Discussion

### Summary of findings and discussion of results

This appears to be the first systematic review on the use of PP in chiropractic practice. We noted that the most frequently studied topic was wellness. Regardless the type of PP (musculoskeletal prevention, public health, or wellness) the proportion of patients who actually consulted specifically for PP was much smaller than the proportion of chiropractors offering the various types of PP, which in turn, in general, was smaller than the proportion of chiropractors who stated that they had a positive opinion on the various types of PP.

More specifically, positive opinions and attitudes to PP were revealed by the majority of chiropractors for both public health activities and wellness, whereas this question was not studied in relation to musculoskeletal prevention. Not surprisingly, almost all surveyed chiropractors offered some type of PP for musculoskeletal disorders and more than half stated that they did so in the public health area but also for wellness.

Although, for all types of PP, it was rarely stated to be the reason for consulting, it could of course have been dealt with somehow through the treatment course, in relation to issues other than those causing the initial reason for consulting.

To simplify the interpretation of these results, the three levels of approach [(i) opinion, (ii) use of service, and (iii) reason for consulting] in relation to the three types of PP [(i) musculoskeletal, (ii) public health, and (iii) wellness] have been illustrated in Table 7.

**Table 7:** Schematic illustration of opinions and use of primary prevention in chiropractic practice

Percent	Prevention of MSK disorders			Public Health										Wellness		
	Opi	Use	RfC	Opinions					Use of service				RfC	Opi	Use	RfC
				A	B	C	D	E	A	B	C	D	E			
90–100%		X X		X	X X			X^a^		X		X		X X	X	
80 – 89%		X					X		X X	X X						
70 – 79%		X							X X			X				
60 – 69%						X X					X X					
50 – 59%							X				X	X			X	
40 – 49%												X			X	
30 – 39%																
20 – 29%																X
10 – 19%			X X										X	X		X X X
0 – 9%			X X X											X		X X X

Opi: Opinions/Use: use of service/RfC: Reasons for Consulting

^a^Of several estimates available only the highest is presented

A: Prescription of dietary supplements or advice on nutrition

B: Prescription of/advice on physical activity

C: Advice on tobacco cessation

D: Detection of skin lesion

E: Non-specific public health

We found it surprising that so few patients feel that chiropractors have something to offer in this area, although the chiropractic profession is encouraged to participate in preventive activities and clearly is interested to do so [42]. The reasons for this need to be explored. Are the reasons that patients, in general, consider chiropractors as belonging to a profession that treats their back problems only, or is it because what is offered is perceived as irrelevant or useless, or is it simply due to lack of information on the subject? Another question is, do chiropractors have the knowledge and skills to perform PP? In addition, it is also important to base PP on facts; what advice and treatments are available to perform PP of musculoskeletal disorders and is chiropractic care really capable of improving the feeling of general well-being, to prevent disease, and improve longevity?

### Methodological considerations of the reviewed studies

#### Quality scores

The quality of studies varied. We classified ten of the studies as being of good quality. On the other hand, we removed six studies from the reporting of results, considering their findings to be uncertain because of their methodological approach. However, they are presented in the checklists, making it possible for interested readers to consult their characteristics and results. Interestingly, we did not note a gradual improvement of the quality scores by year of study, indicating that research teams did not learn from each other’s ‘mistakes’. The methodological approach seemed to be an aspect that was inherent in the individual research teams.

#### Definitions of primary prevention

Our review was somewhat limited from the lack of specific definitions of PP in most studies, which could have resulted in misclassifications, in particular in relation to wellness. We did not feel it fair to include a quality criterion on this issue unless the main topic of the survey was prevention, but even when this was the primary aim of the study, the descriptions of PP were vague and did not allow us to contextualise with accuracy. This could make it difficult to decide whether study subjects and/or the research teams had a clear opinion of whether they really dealt with PP (i.e. the prevention of a condition in healthy subjects) or if they mixed it up with other types of prevention, such as prevention of recurrences or perhaps even maintenance care and also whether the activity related to public health in general or not. These problems could have been resolved if survey instruments and the specific questions had been available, but this was often not the case. However, often the context and surrounding information could remedy this weakness, such as when authors mentioned that they studied the subluxation and its link to disease, which would indicate that chiropractors endorsing this concept considered it possible to perform PP through chiropractic adjustments.

#### Low response rates

Another problem in the literature that made our interpretations difficult was that the response rates were (as is often the case in surveys) mainly low (below 80%) and that only few authors compared responders to non-responders. This probably (but not for sure) limits the representativeness of the study samples, assuming that there is heterogeneity among chiropractors and their patients on these issues. Although it is impossible to define a cut-point for when a response rate is too low to result in generalizability, perhaps authors and editors should consider whether surveys with response rates as low as 10% and less are worthy of reporting in the literature. Stating this, it is acknowledged that the 10% response rate cut off used in this review was arbitrarily chosen.

### Methodological considerations of own survey

In relation to the various methodological considerations surrounding this review, our work was guided by a modified AMSTAR checklist [16]. One of our reviewers is experienced in performing systematic reviews and two of the reviewers are chiropractors with an insight in the concepts and jargon of this field. The systematic approach in this type of review limits but does not remove the subjective approach to data analysis and interpretation. It is possible that another team could have used other inclusion and exclusion criteria, selected a different methodological approach, or interpreted the data differently, but as the two blind reviewers agreed on every point in this review and the referee was never needed we could conclude that our approach was at least user-friendly.

However, it is possible that we failed to retrieve some relevant surveys on this topic. In the chiropractic field, some professional journals exist that are ‘invisible’ when searching through the usual library sources. We initially searched two medical databases (PubMed and Embase) later completed with Index to Chiropractic Literature and Google Scholar. This approach added two articles, but we could have missed out on some other relevant work, assuming that they could have been traceable through other library databases.

As we did not explicitly search the grey literature, we would have missed surveys published by such media but, probably, studies not published through the peer-review process would have a relatively low methodological standard, which would limit their usefulness. For this review, we were unable to obtain three of the articles found through the literature search, which, potentially, were lost from the review. Nevertheless, it is unlikely that (at the most) three additional articles would have markedly changed our findings.

## Conclusions

Interestingly, according to this review of the chiropractic literature, more research efforts have been put into wellness than into prevention of musculoskeletal disorders or public health-related disorders such as cardiovascular disease. It therefore seems that parts of the chiropractic profession are in search of an understanding of various aspects of clinical practice over and above its traditional musculoskeletal role.

Although it is possible that PP is provided as a natural element during the course of treatment – and hence not discovered through surveys asking for reason for consulting, it is clear that only a small proportion of chiropractic patients consult for PP, despite the readiness of the profession to offer such services.

## Future directions

If chiropractors wish to provide more PP to their patients, it would be necessary to review the literature on the effectiveness of this approach in relation to musculoskeletal prevention and wellness. It is quite possible that this will reveal a dearth of relevant information, which in turn should incite interested chiropractors to encourage well designed clinical studies on these topics.

## Abbreviations

PP: Primary Prevention
